# Metabolic Syndrome in Psychotic Disorder Patients Treated With Oral and Long-Acting Injected Antipsychotics

**DOI:** 10.3389/fpsyt.2018.00744

**Published:** 2019-01-16

**Authors:** Antonio Ventriglio, Ross J. Baldessarini, Giovanna Vitrani, Iris Bonfitto, Angela Chiara Cecere, Angelo Rinaldi, Annamaria Petito, Antonello Bellomo

**Affiliations:** ^1^Department of Clinical and Experimental Medicine, University of Foggia, Foggia, Italy; ^2^International Consortium for Psychotic & Mood Disorder Research, McLean Hospital, Belmont, MA, United States; ^3^Department of Psychiatry, Harvard Medical School, McLean Hospital, Boston, MA, United States

**Keywords:** metabolic syndrome, antipsychotics, long-acting injected, schizoaffective, schizophrenia

## Abstract

**Background:** Severe mental illnesses are associated with increased risks for metabolic syndrome (MetS) and other medical disorders, often with unfavorable outcomes. MetS may be more likely with schizoaffective disorder (SzAff) than schizophrenia (Sz). MetS is associated with long-term antipsychotic drug treatment, but relative risk with orally administered vs. long-acting injected (LAI) antipsychotics is uncertain.

**Methods:** Subjects (*n* = 151 with a DSM-IV-TR chronic psychotic disorder: 89 Sz, 62 SzAff), treated with oral or LAI antipsychotics were compared for risk of MetS, initially with bivariate comparisons and then by multivariate regression modeling.

**Results:** Aside from measures on which diagnosis of MetS is based, factors preliminarily associated with MetS included antipsychotic drug dose, “high-risk” antipsychotics associated with weight-gain, older age and female sex. Defining factors associated with diagnosis of MetS ranked in multivariate regression as: higher fasting glucose, lower LDL cholesterol, higher diastolic blood pressure, and higher BMI. Risk of MetS with antipsychotics ranked: quetiapine ≥ clozapine ≥ paliperidone ≥ olanzapine ≥ risperidone ≥ haloperidol ≥ aripiprazole. Other associated risk factors in multivariate modeling ranked: higher antipsychotic dose, older age, and SzAff diagnosis, but not oral vs. LAI antipsychotics

**Conclusions:** SzAff diagnosis and higher antipsychotic doses were associated with MetS, whereas orally vs. injected antipsychotics did not differ in risk of MetS.

## Introduction

Persons with severe mental illnesses have increased risk for metabolic disorders, including metabolic syndrome (MetS), characterized by obesity, type 2 diabetes mellitus, dyslipidemia, and hypertension (1). Such disorders appear to be related to an unhealthy diet, lack of regular exercise, adverse effects of psychotropic drugs, and possibly to undefined risk factors associated with the illnesses themselves (2, 3). Much of the research on this topic has involved patients diagnosed with chronic psychotic or mood disorders, particularly schizophrenia (Sz) and bipolar disorder (2, 4–6). Few studies have compared physical health of subjects diagnosed with schizoaffective disorder (SzAff) to that of other patients with other psychotic-disorder diagnoses, including Sz, but SzAff patients may have a greater risk of MetS than those with other major psychiatric disorders (6). SzAff patients are characterized by emotional and behavioral instability over time as well as psychotic features, and often are treated with relatively complex pharmacological regimens (7). Both emotional instability and complex treatments may contribute to an increased risk of metabolic disorders (1).

Also uncertain is whether specific types of medicines differ appreciably in their associations with risks of metabolic disorders. In particular, the extent to which relative metabolic risks of modern or second-generation antipsychotics (SGAs) and their long-acting injected (LAI) preparations differ from older or orally administered antipsychotics remains uncertain (1, 8–10).

The preceding considerations led us to compare clinical measures, in particular indices of metabolic health, among SzAff vs. Sz patient-subjects to identify factors associated specifically with MetS, including comparison of orally administered vs. LAI antipsychotics. We hypothesized that SzAff subjects would have a higher risk of MetS than Sz subjects, and that the risk might be lower with LAI antipsychotic treatments.

## Methods

From June 2014 to February 2017, we enrolled study subjects as part of a program monitoring the health of psychotic disorder patients attending the Day Hospital Service for Severe Mental Disorders in the Psychiatric Department at the University of Foggia Medical Center. A total of 151 consecutive patients were enrolled as study-subjects, including 89 diagnosed with Sz and 62 as SzAff by two expert clinicians (AB, AV) based on DSM-IV-TR (Diagnostic and Statistical Manual of mental disorders- Text Revision) criteria (11). Treatments were selected clinically and included oral antipsychotics (*n* = 64, with or without mood-stabilizers or antidepressants) as well as LAI antipsychotics (*n* = 87, usually as monotherapy).

All subjects provided written informed consent to participate, after study procedures approved by the University of Foggia medical center ethics committee were explained to them. Patients were enrolled in a stable phase of their illness and treatments; candidates who required psychiatric hospitalization, had revised treatment protocols within the previous 6 months, were actively abusing alcohol or drugs (confirmed by urine assays), or were pregnant, were excluded from the study.

Current psychiatric morbidity was assessed and rated with the Positive and Negative Syndrome Scale (PANSS) (12), and Brief Psychiatric Rating Scale (BPRS) (13) by two experienced psychiatrist-investigators (AB, AV). Raters were held unaware of treatments given, and their ratings yielded high, independent, interrater agreement (χ^2^ ≥ 0.90). Being considered “mildly ill” corresponded to a PANSS total score of ≤58 or BPRS score of ≤31, “moderately ill” corresponded to PANSS ratings of 59–75 or BPRS scores of 32–40, “moderately severely ill” corresponded to PANSS of 76–95 or BPRS of 41–53, and “severely ill” corresponded to a PANSS of 96–116 or BPRS of 54–126 (12, 13).

We also collected data on: demographics (sex, age, employment status), current pharmacological treatments (oral or LAI antipsychotics, mood stabilizers [MSs], and antidepressants [ADs]), and their doses; anthropometric and metabolic measures (height [cm] and weight [kg] for body-mass index [BMI]), systolic and diastolic blood pressure (mm Hg), pulse (beats/min); serum concentrations of fasting glucose (FBS; mg/dL), %-glycated hemoglobin (Hgb-A1c), total cholesterol (mg/dL), low density lipoproteins (LDL; mg/dL), high density lipoproteins (HDL; mg/dL), triglycerides (mg/dL); waist circumference (cm), electrocardiographic rate-corrected QT repolarization interval (QTc, msec); serum levels of prolactin (ng/dL), thyroid stimulating hormone (TSH, mIU/L), and free thyroxin and triiodothyronine. We also recorded adverse events associated with treatment, and rated treatment-adherence with the 30-item Drug Attitude Inventory (DAI-30) (14).

We rated subjects for the presence of MetS defined by current, revised International Diabetes Federation (IDF) criteria, American Heart Association and International Association for the Study of Obesity (15, 16). MetS required meeting ≥3 of the following 5 criteria: [a] large waist circumference (≥102 cm in men, ≥88 cm in women); [b] elevated serum triglycerides (≥150 ng/dL); [c] low HDL-cholesterol (<40 mg/dL in men and <50 in women); high blood pressure (≥130 mm Hg systolic or ≥85 mm diastolic); elevated glucose as fasting blood sugar (FBS >100 mg/dL).

To facilitate comparisons, we converted antipsychotic doses to approximate oral daily mg-chlorpromazine-equivalents (CPZ-eq); LAI antipsychotic doses were estimated as total mg doses per days of injection cycles for conversion to CPZ-eq (17, 18). For MSs, we converted dosages to approximate daily mg-equivalents of lithium carbonate (Li-eq) (18, 19). Antidepressants were noted as being prescribed or not.

We compared measures collected among subjects diagnosed with SzAff and Sz, treated with LAI and oral antipsychotics, emphasizing comparisons of subjects with vs. without MetS. Data analyses used commercial statistical programs (Statview-5, SAS Corp., Cary, North Carolina, USA for spreadsheets; Stata.13.0, Stata Corp., College Station, Texas, USA). Data are presented as means ± standard deviation (SD) or with 95% confidence intervals (CI), or as percentages (%), unless stated otherwise. Continuous data were compared using nonparametric Mann-Whitney rank-sum test (*z*-score) to avoid problems of non-normal distribution of values, and categorical data were tested with contingency tables (χ^2^). Factors yielding *p* < 0.10 in preliminary bivariate comparisons were considered in multivariate logistic regression modeling, with presence of MetS as the outcome measure.

## Results

### Sample Characteristics and Treatments

The 151 patient-subjects were aged 42.1 ± 12.4 years; 52.9% were men, 18.5% were employed. Diagnoses included Sz (*n* = 89; 58.9%) and SzAff (*n* = 62; 41.1%). More men than women were diagnosed with Sz (χ^2^ = 6.76; *p* = 0.009). Treatments included oral antipsychotics in 42.3%, and LAI antipsychotics in 57.7% (none received both). Antipsychotics were combined with mood-stabilizers (MSs) in only 14.5%, or with antidepressants (ADs) in 12.3% (ranking by use: duloxetine > paroxetine > citalopram or *S*-citalopram > sertraline). Both adjunctive treatments were given selectively with oral antipsychotics, by 6.4- (MSs) or 7.0-times more (ADs; both *p* ≤ 0.006) among SzAff than Sz subjects. Antipsychotic doses averaged 313 ± 329 mg/day CPZ-eq, and MS (carbamazepine, lithium carbonate, sodium valproate) total daily Li-eq doses averaged 650 ± 244 mg. Overall, clinical ratings averaged 75.0 ± 34.7 for PANSS and 51.6 ± 23.6 for BPRS; both indicate moderate symptomatic severity, even though all patients reported clinical and treatment stability for at least six continuous preceding months. Prolonged, stable dosing assured that even the LAI antipsychotics were at pharmacokinetic steady-state.

Subjects who received LAI vs. oral antipsychotics had significantly lower levels of symptomatic morbidity. PANSS scores were, respectively, 58.0 ± 27.6 vs. 98.1 ± 29.9, and BPRS scores averaged 40.1 ± 15.0 vs. 67.1 ± 24.5 (*z*-scores = 7.76 and 6.85, both *p* < 0.0001).

No subject was considered to have a substance-use disorder, as was supported by urine drug assays, consistent with current substance abuse as an exclusion criterion. Adherence to prescribed treatments was considered good, as supported by a DAI-30 score of 9.64 ± 2.19 (of a total maximum of 30). There was a moderate rate of reported, treatment-associated, adverse events (19.8%), most of which involved motor slowing or mild tremor.

### Risk and Measures Associated With Metabolic Syndrome

Of the entire sample, 31.8% met diagnostic criteria for MetS (Table 1): 42.3% of women and 22.5% of men. Overall, BMI averaged 27.7 ± 5.72 kg/m^2^, in the nearly obese range. However, 35.8% of subjects (38.0% of women and 33.8% of men) had BMI of ≥28.8 kg/m^2^, taken to indicate obesity (15, 16).

**Table 1:** Factors associated with metabolic syndrome in 151 psychotic disorder patients.

**Factor**	**Metabolic syndrome**		**Statistic**	***p*-value**
	**Present**	**Absent**	**(*z* or χ^2^)^a^**	
All cases (*N* = 151)	31.8 [24.5–39.9]	68.2 [60.1–75.5]	–	–
Women (%)	62.5 [47.4–76.0]	39.8 [30.3–49.9]	6.77	0.009
Older age (years)	45.0 [42.0–48.0]	40.8 [38.2–43.4]	2.18	0.03
Unemployed (%)	83.3 [69.8–92.5]	80.6 [71.6–87.7]	0.67	0.69
Schizoaffective diagnosis (%)	52.1 [37.2–66.7]	35.9 [26.7–46.0]	3.53	0.06
**Psychosis**				
BPRS score	57.1 [49.8–64.4]	49.0 [44.6–53.4]	1.96	0.05
PANSS score	81.7 [71.1–92.3]	71.9 [65.3–78.5]	1.55	0.12
Substance abuse (%)	4.17 [0.51–14.3]	1.94 [0.24–6.84]	0.63	0.43
High-risk antipsychotics (%)^b^	29.2 [17.0–44.1]	14.6 [3.39–22.9]	4.50	0.03
LAI antipsychotics (%)	50.0 [35.2–64.8]	50.0 [35.2–64.8]	1.67	0.20
Antipsychotic dose (CPZ-eq, mg/day)	423 [266–580]	261 [235–287]	2.14	0.004
Treatment adherence (DA130)	9.95 [9.25–10.7]	9.59 [9.12–10.1]	0.86	0.39
Adverse drug effects (%)	22.9 [12.0–37.3]	18.4 [11.5–27.3]	0.41	0.52
Mood-stabilizers given (%)	18.8 [8.95–32.6]	12.6 [6.89–20.6]	0.99	0.32
Antidepressants given (%)	2.08 [0.05–11.1]	6.80 [2.28–13.5]	1.45	0.23
BMI (kg/m^2^)	30.3 [28.3–32.3]	26.6 [25.7–27.5]	3.43	0.0006
Obesity (% BMI≥28.8)	64.6 [49.5–77.8]	22.3 [14.7–31.6]	25.4	<0.0001
Waist circumference (cm)	117 [110–124]	105 [100–110]	2.74	0.006
Systolic BP (mm Hg)	120 [117–123]	116 [114–117]	2.28	0.02
Diastolic BP (mm Hg)	76.5 [75.5–81.5]	73.7 [71.7–75.8]	2.58	0.01
Pulse rate (per min)	85.0 [82.7–86.1]	84.4 [82.4–87.6]	0.37	0.71
ECG repolarization (QTc, msec)	409 [401–417]	407 [403–411]	0.13	0.90
Glucose (FBS, mg/dL)	105 [99.6–110]	88.8 [87.2–90.4]	4.91	<0.0001
HgbA1c (%)	5.95 [5.71–6.19]	5.62 [5.49–5.75]	2.79	0.005
Total cholesterol (mg/dL)	217 [206–228]	200 [193–207]	2.50	0.01
LDL cholesterol (mg/dL)	161 [148–174]	140 [132–148]	2.75	0.006
HDL cholesterol (mg/dL)	42.9 [40.3–45.5]	50.2 [47.9–52.5]	3.86	0.0001
Triglycerides (mg/dL)	204 [181–227]	147 [135–159]	4.31	0.0002
TSH (nU/L)	2.56 [2.07–3.05]	2.14 [1.89–2.39]	1.65	0.10
Free thyroxin (ng/dL)	1.13 [1.07–3.05]	1.30 [1.09–1.51]	1.09	0.27
Free triiodothyronine (ng/dL)	0.358 [0.340–0.376]	0.353 [0.340–0.366]	0.86	0.39
Prolactin (ng/dL)	42.9 [41.4–54.5]	50.2 [47.1–65.9]	1.53	0.12

*BPRS, Brief Psychiatric Rating Scale; PANSS, Positive and Negative Syndrome Scale; LAI, long-acting injectable antipsychotics; CPZ, chlorpromazine; DAI, Drug Attitude Inventory; BMI, body-mass index; BP, blood pressure; ECG, electrocardiogram; Hgb, hemoglobin; LDL, low-density lipoprotein; HDL, high-density lipoprotein; TSH, thyroid-stimulating hormone*.

a *Mann-Whitney (z-score) or contingency table (χ^2^)*.

b *Clozapine, olanzapine, paliperidone, quetiapine*.

Other factors possibly associated with MetS included: female sex, older age, SzAff vs. Sz diagnosis, higher BPRS psychosis score (which was associated with greater APD doses: Spearman *r*_*s*_ = 0.252, slope = 1.68 [0.653–2.72], *p* = 0.002), treatment with antipsychotics with relatively high risk of obesity and MetS (clozapine, olanzapine, paliperidone, quetiapine; Table 2), higher CPZ-eq antipsychotic dose, but not orally administered vs. LAI antipsychotics (Table 1). Additional metabolic and cardiovascular measures did not differ between subjects with vs. without MetS, including assays of TSH and thyroid hormones, prolactin, pulse rate, and ECG repolarization interval (QTc), nor did BPRS or PANSS ratings of psychosis-severity differ (Table 1).

**Table 2:** Multivariable logistic regression modeling: diagnostic measures associated with metabolic syndrome.

**Measure**	**OR [95% CI]**	**χ^**2**^**	***p*-value**
Higher FBS	1.12 [1.07–1.17]	25.2	<0.0001
Lower HDL	1.16 [1.08–1.24]	17.0	<0.0001
Diastolic blood pressure	1.10 [1.04–1.16]	10.3	0.001
Higher BMI	1.17 [1.06–1.29]	9.53	0.002
Female sex	5.72 [1.85–17.7]	9.21	0.002

*Model fit χ^2^ = 93,5, p < 0.0001. Not associated, age, LDL level, waist-circumference*.

As expected, measures that contributed to its diagnosis were highly deviant among subjects with MetS, including obesity, waist circumference, blood pressure, FBS, hemoglobin glycation, serum concentrations of cholesterol (higher total and LDL, lower HDL) and triglycerides (Table 1). BMI was not used to define MetS but was markedly elevated in subjects with MetS (Table 1). We also tested the strengths of association of such measures with the diagnosis of MetS using logistic regression modeling (Table 2). These ranked as: higher FBS ≥ lower HDL ≥ higher diastolic blood pressure ≥ higher BMI ≥ female sex (BMI and sex were not included in diagnostic criteria for MetS).

### Treatments and Metabolic Syndrome

LAI antipsychotics were more prescribed than oral agents (57.7% vs. 42.3%), particularly among Sz vs. SzAff subjects (65.5% vs. 34.5%; χ^2^ = 3.67, *p* = 0.055), whereas oral agents were used in half of both diagnostic groups. Average CPZ-eq daily doses of oral and LAI antipsychotics among Sz (295 mg) and SzAff subjects (338 mg) did not differ significantly. LAI paliperidone palmitate was the most prescribed antipsychotic agent in both diagnostic groups (Sz 34.8%; SzAff 27.4%; 31.8% overall); other LAI antipsychotic usage ranked: risperidone extended release (10.6%) > aripiprazole long acting (9.28%) > olanzapine palmitate (4.64%). Usage of oral antipsychotics ranked: risperidone (Sz 11.2%, SzAff 12.9%, 11.9% overall) > haloperidol (7.28% overall) = olanzapine (7.28%) > aripiprazole (5.30%) > quetiapine (3.98%) ≥ paliperidone (3.31%) = clozapine (3.31%) > ziprasidone 1.32%.

Several metabolic measures were somewhat more favorable with use of LAI vs. oral antipsychotics, including total cholesterol (192 vs. 223 mg/dL), LDL cholesterol (125 vs. 175 mg/dL), triglycerides (148 vs. 188 mg/dL); waist circumference (103 cm [40.6 in] vs. 117 cm [46.1 in]); the cardiac QTc repolarization interval (399 vs. 413 msec); and circulating prolactin (42.7 vs. 61.3 ng/dL).

We compared the prevalence of MetS among subjects treated with different antipsychotic agents. Relatively high-risk drugs were quetiapine (83.3%), clozapine (60.0%), paliperidone (34.0%) and olanzapine (33.4%; Table 3). Of note, these risks were not accounted for by dose as prevalence of MetS and CPZ-eq doses were not significantly correlated (Table 3).

**Table 3:** Risk of metabolic syndrome: antipsychotic agents and doses.

**Drug**	**Metabolic Syndrome**	**Daily dose**
	**Prevalence (% [CI])**	**(CPZ-eq mg [CI])**
Quetiapine	83.3 [35.9–99.6]	229 [89.9–368]
Clozapine	60.0 [14,7–94.7]	615 [196–1034]
Paliperidone	34.0 [21.5–48.3]	298 [265–330]
Olanzapine	33.4 [13.3–59.0]	378 [309–448]
Risperidone	23.5 [10.7–41.2]	266 [232–301]
Haloperidol	18.2 [2.28–51.8]	259 [197–321]
Aripiprazole	18.2 [5.19–40.3]	121 [95.4–146]

*Risk of MetS is not significantly associated with CPZ-eq dose (Spearman r_s_ = 0.402, p = 0.325), but the drugs differ overall (χ^2^ = 13.4, p = 0.04). Relatively high-risk drugs are quetiapine, clozapine, paliperidone and olanzapine*.

### Multivariable Modeling: Factors Associated With Metabolic Syndrome

We used multivariable logistic regression modeling to identify factors associated independently with MetS. In order of significance, associated factors ranked: CPZ-eq antipsychotic dose, older age, and SzAff > Sz diagnosis, but not oral vs. LAI antipsychotics (Table 4).

**Table 4:** Multivariable logistic regression modeling: risk factors associated with metabolic syndrome.

**Factor**	**OR [95% CI]**	**χ^2^**	***p*-value**
Antipsychotic dose (CPZ-eq)	1.003 [1.001–1.005]	4.80	0.028
Older age	1.03 [1.01–1.07]	4.76	0.029
Diagnosis: Schizoaffective	2.28 [1.06–4.90]	4.46	0.035
Oral vs. LAI antipsychotics	1.01 [0.46–2.24]	0.001	0.98

*Model fit χ^2^ = 17.9, p = 0.001. Not associated, sex, diagnosis, psychosis severity. (BPRS rating)*.

## Discussion

This study involved 151 patient-subjects with chronic psychotic disorders who had been clinically stable on constant medication regimens for at least 6 months. LAI antipsychotics were given to 57.7%, and oral agents to 42.3%. LAI agents were more often given to Sz subjects, whereas use of oral antipsychotics was similarly prevalent in both SzAff and Sz subjects. SzAff subjects were also 6–7-times more likely to be given co-treatment with a mood-stabilizer or antidepressant. All subjects were rated at moderate symptomatic severity by PANSS and BPRS. Adherence to prescribed treatments was rated as good by DAI-30 score, and the risk of adverse effects was moderate at 19.8%.

The overall prevalence of MetS (31.8%) was moderate, but ranged from 18.2% with aripiprazole to 83.3% with quetiapine (Table 3). By comparison, reported prevalence of MetS in patients with psychotic disorders has ranged from 30 to 67% (1, 7, 20, 21). Based on multivariate modeling, factors associated with MetS were antipsychotic dose, older age, and SzAff diagnosis, but not oral vs. LAI antipsychotics.

Of note, the present finding of significantly greater risk of MetS with SzAff vs, Sz diagnoses supports one hypothesis of this study, and adds to a previous suggestion of such a relationship (6). Although use of complex medication regimens was much more prevalent among SzAff subjects, these were infrequent and not significantly associated with risk of MetS. In addition, CPZ-eq antipsychotic doses were somewhat higher among SzAff subjects, although both factors appeared to operate somewhat independently (Table 4). Nevertheless, we suggest that the relative instability of SzAff disorders contributes to the use of more complex treatments (7) and this feature as well perhaps as intrinsic characteristics of such patients may contribute to risk of MetS.

In addition, contrary to prediction, we did not find a significant difference in risk of MetS in association with treatment with oral vs. LAI antipsychotics. This finding appears to be consistent with other recent reports indicating that LAI agents may not be safer than oral antipsychotics (8, 10). However, several measures tended to be less abnormal with LAI treatments (including lower total and LDL cholesterol, triglycerides and prolactin, smaller waist-circumference, and shorter QTc interval). In addition, we found marked differences in risk of MetS between particular antipsychotic agents, with higher risk associated with quetiapine, clozapine, paliperidone, and olanzapine, which are known to be associated with relatively high risks of weight-gain (1, 2, 18, 22, 23).

### Limitations

The study included a relatively small number of subjects and its findings may not generalize to other sites. Its cross-sectional design supports associations with MetS, but precludes causal inferences. In addition, estimates of CPZ-eq doses of LAI antipsychotics are not adjusted for probable but uncertain differences in bioavailability of injected vs. orally administered drugs.

## Conclusions

This observational study of 151 patient-subjects with chronic psychotic disorders found a moderate prevalence of MetS (31.8%), which was associated with being overweight or obese and with antipsychotic agents prone to leading to weight-gain, as well as higher antipsychotic CPZ-eq doses, and older age. Notably, risk of MetS was somewhat greater among SzAff than Sz subjects, but did not differ significantly between treatment with oral and LAI antipsychotics nor with the relatively infrequent use of adjunctive mood-stabilizers or antidepressants. However, several metabolic measures tended to be less abnormal among SzAff than Sz subjects. The association of MetS with SzAff (more than with Sz) probably reflects the complexity of SzAff disorders and their pharmacological treatment, including somewhat higher antipsychotic doses and more co-treatment with mood-stabilizers and antidepressants, but may also reflect other unknown characteristics of the disorders themselves.

## Author Contributions

AV, GV, IB, AC, AR, and AP recruited patients and collected data. AV and RB wrote the paper. AB supervised clinical work and the manuscript drafting.

### Conflict of Interest Statement

The authors declare that the research was conducted in the absence of any commercial or financial relationships that could be construed as a potential conflict of interest.
